# 2-(2,4-Di­fluoro­phen­yl)-4,5-dimethyl-1-(4-methyl­phen­yl)-1*H*-imidazole monohydrate

**DOI:** 10.1107/S1600536813026354

**Published:** 2013-10-02

**Authors:** Natesan Srinivasan, Syed Rafee Ahamed Rizwana Begum, Ramu Hema, Balasubramanian Sridhar, Azhagan Ganapathi Anitha

**Affiliations:** a Department of Chemistry, S.K.P. Engineering College, Thiruvanamalai 606 611, India; b Department of Physics, Seethalakshmi Ramaswami College (Autonomous), Tiruchirappalli 620 002, India; c Department of Physics, K. Ramakrishnan College of Engineering, Samayapuram, Tiruchirappalli 621 112, India; d Laboratory of X-ray Crystallography, Indian Institute of Chemical Technology, Hyderabad 500 007, India

## Abstract

The asymmetric unit of the title compound, C_18_H_16_F_2_N_2_·H_2_O, contains two independent mol­ecules (*A* and *B*), and two independent water mol­ecules of crystallization. In mol­ecule *A*, the imidazole ring makes dihedral angles of 47.46 (7) and 60.98 (6)° with the 2,4-di­fluoro­phenyl and methyl­phenyl rings, respectively. The corresponding angles in mol­ecule *B* are 45.85 (7) and 62.78 (7)°, respectively. The dihedral angle between the two benzene rings is 64.98 (7)° in mol­ecule *A* and 65.53 (7)° in mol­ecule *B*. In the crystal, the two independent mol­ecules are linked by O—H⋯N and O—H⋯O hydrogen bonds, forming chains propagating along [100]. These chains are linked *via* C—H⋯F hydrogen bonds, forming slab-like two-dimensional networks lying parallel to (001).

## Related literature

For background and the biological properties of imidazole derivatives, see: Dutta *et al.* (2009 ▶); Hori *et al.* (2000 ▶); Khabnadideh *et al.* (2003 ▶); Mamolo *et al.* (2004 ▶); Quattara *et al.* (1987 ▶); Sengupta & Bhattacharya (1983 ▶); Ucucu *et al.* (2001 ▶); Noilada *et al.* (2004 ▶). For related structures, see: Rizwana *et al.* (2013 ▶); Gayathri *et al.* (2010 ▶); Rosepriya *et al.* (2011 ▶).
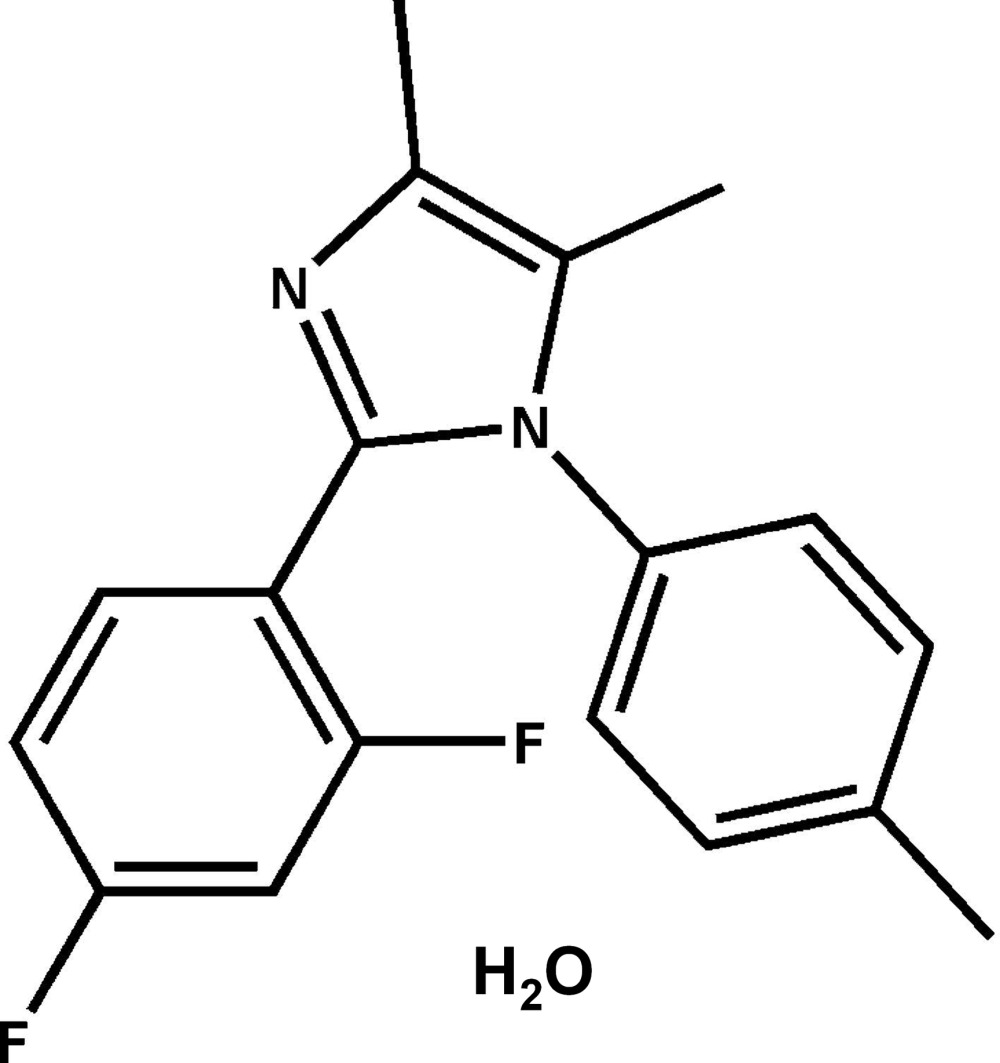


## Experimental

### 

#### Crystal data

C_18_H_16_F_2_N_2_·H_2_O
*M* *_r_* = 316.34
Triclinic, 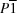

*a* = 7.9424 (9) Å
*b* = 14.5238 (16) Å
*c* = 14.6561 (16) Å
α = 75.087 (2)°
β = 89.990 (2)°
γ = 86.012 (2)°
*V* = 1629.5 (3) Å^3^
*Z* = 4
Mo *K*α radiation
μ = 0.10 mm^−1^
*T* = 294 K
0.30 × 0.25 × 0.20 mm

#### Data collection

Bruker SMART APEX CCD area-detector diffractometer
18763 measured reflections
7545 independent reflections
5782 reflections with *I* > 2σ(*I*)
*R*_int_ = 0.026

#### Refinement

*R*[*F* ^2^ > 2σ(*F* ^2^)] = 0.048
*wR*(*F* ^2^) = 0.140
*S* = 1.00
7545 reflections
433 parameters
13 restraints
H atoms treated by a mixture of independent and constrained refinement
Δρ_max_ = 0.26 e Å^−3^
Δρ_min_ = −0.26 e Å^−3^

### 

Data collection: *SMART* (Bruker, 2001 ▶); cell refinement: *SAINT* (Bruker, 2001 ▶); data reduction: *SAINT*; program(s) used to solve structure: *SIR92* (Altomare *et al.*, 1994 ▶); program(s) used to refine structure: *SHELXL97* (Sheldrick, 2008 ▶); molecular graphics: *ORTEP-3 for Windows* (Farrugia, 2012 ▶); software used to prepare material for publication: *SHELXL97* and *PLATON* (Spek, 2009 ▶).

## Supplementary Material

Crystal structure: contains datablock(s) I, global. DOI: 10.1107/S1600536813026354/su2650sup1.cif

Structure factors: contains datablock(s) I. DOI: 10.1107/S1600536813026354/su2650Isup2.hkl

Supplementary material file. DOI: 10.1107/S1600536813026354/su2650Isup3.cml

Additional supplementary materials:  crystallographic information; 3D view; checkCIF report

## Figures and Tables

**Table 1:** Hydrogen-bond geometry (Å, °)

*D*—H⋯*A*	*D*—H	H⋯*A*	*D*⋯*A*	*D*—H⋯*A*
O1*W*—H1*WA*⋯N5^i^	0.82 (3)	2.17 (3)	2.987 (2)	177 (2)
O1*W*—H1*WB*⋯O1*W* ^ii^	0.72 (2)	2.42 (2)	2.894 (3)	125 (2)
O2*W*—H2*WA*⋯N3	0.84 (3)	2.16 (3)	2.995 (2)	174 (2)
O2*W*—H2*WB*⋯O2*W* ^iii^	0.73 (2)	2.42 (2)	2.910 (2)	126 (2)
C17—H17⋯F3^iv^	0.93	2.46	3.253 (2)	143

Symmetry codes: (i) 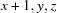; (ii) 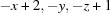; (iii) 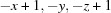; (iv) 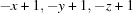.

**Fig. 1.**
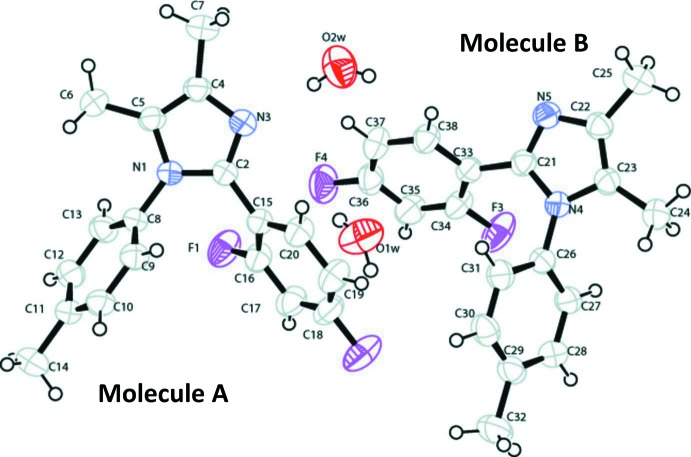
The molecular structure of the two independent molecules (A and B) of the title compound, with atom labelling. Displacement ellipsoids are drawn at the 50% probability level.

**Fig. 2.**
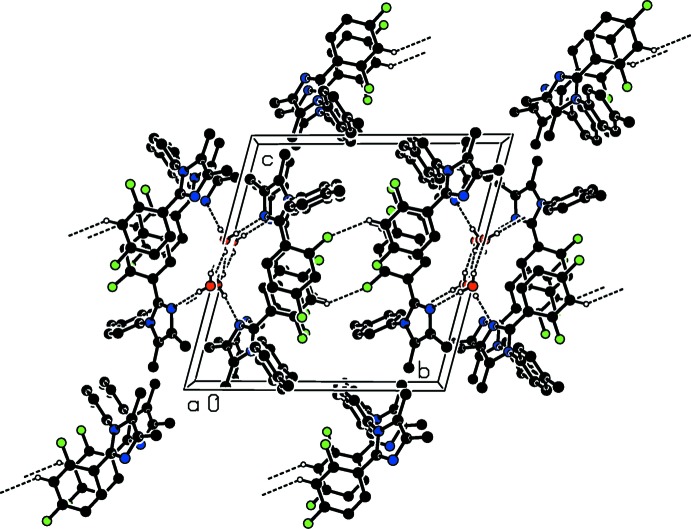
The crystal packing of title compound viewed along the axis. The hydrogen bonds are shown as dashed lines (see Table 1 for details; H atoms not involved in hydrogen bonding have been omitted for clarity).

## supplementary crystallographic information

### 1. Comment

Imidazole derivatives have a wide range of biological properties, such as antifungal (Hori *et al.*, 2000; Mamolo *et al.*, 2004) and antibacterial (Khabnadideh *et al.*, 2003) activities. They are well known analgesic (Ucucu *et al.*, 2001), anti-inflammatory (Noilada *et al.*, 2004), anthelmintic (Dutta *et al.*, 2009), antiparasitic (Quattara *et al.*, 1987), as well as antimicrobial (Sengupta & Bhattacharya, 1983) agents. In view of the interesting biological and pharmacological activities of Imidazole derivatives, the title compound was synthesized and its crystal structure is reported on herein.

The asymmetric unit of the title compound, Fig. 1, consists of two independent molecules (A and B) and two water molecules. In molecule A, the imidazole ring makes dihedral angles of 47.46 (7)° and 60.98 (6)° with the 2,4-difluorophenyl (C15-C20) and the methylphenyl rings (C8-C13), respectively, whereas in molecule B, the corresponding angles are 45.85 (7)° (C33-C38) and 62.78 (7)° (C26-C31). The dihedral angle between the two benzene rings is 65.52 (7)° in molecule A and 64.98 (6)° in molecule B.

In the crystal, the two independent molecules are linked by O—H···N and O—H···O hydrogen bonds forming chains propagating along [100]. These chains are linked via C—H···F hydrogen bonds forming slab-like two-dimensional networks lying parallel to the ab plane (Table 1 and Fig. 2).

### 2. Experimental

To pure butane-2,3-dione (1.48 g, 15 mmol) in ethanol (10 ml), *p*-toluidine (1.6 g, 15 mmol), ammonium acetate (1.15 g, 15 mmol) and 2,4-difluorobenzaldehyde (1.1 g, 15 mmol) were added over about 60 min while maintaining the temperature at 333 K. The reaction mixture was refluxed for 7 days and extracted with dichloromethane. The solid that separated was purified by column chromatography using hexane:ethyl acetate (1:1 / v:v) as the eluent [Yield: 1.93 g (46%)]. Slow evaporation of a solution of the title compound in chloroform yielded prism-like colourless crystals suitable for X-ray diffraction analysis.

### 3. Refinement

The water H atoms were located in difference Fourier maps and refined with distance restraints: O-H = 0.82 (2) Å and H···H = 1.35 (2) Å, with *U*_iso_(H) = 1.5*U*_eq_(O). The C bound H atoms were placed in geometrically idealized positions and constrained to ride on their parent atoms: C—H = 0.93 - 0.96 Å with *U*_iso_(H) = 1.5*U*_eq_(C-methyl) and = 1.2*U*_eq_(C) for other H atoms.

### Crystal data

**Table:**

C_18_H_16_F_2_N_2_·H_2_O	*Z* = 4
*M**_r_* = 316.34	*F*(000) = 664
Triclinic, *P*1	*D*_x_ = 1.290 Mg m^−^^3^
Hall symbol: -P 1	Mo *K*α radiation, λ = 0.71073 Å
*a* = 7.9424 (9) Å	Cell parameters from 5735 reflections
*b* = 14.5238 (16) Å	θ = 2–25°
*c* = 14.6561 (16) Å	µ = 0.10 mm^−^^1^
α = 75.087 (2)°	*T* = 294 K
β = 89.990 (2)°	Prism, colourless
γ = 86.012 (2)°	0.30 × 0.25 × 0.20 mm
*V* = 1629.5 (3) Å^3^	

### Data collection

**Table:**

Bruker SMART APEX CCD area-detector diffractometer	5782 reflections with *I* > 2σ(*I*)
Radiation source: fine-focus sealed tube	*R*_int_ = 0.026
Graphite monochromator	θ_max_ = 28.1°, θ_min_ = 1.8°
ω scan	*h* = −10→10
18763 measured reflections	*k* = −18→18
7545 independent reflections	*l* = −19→19

### Refinement

**Table:**

Refinement on *F*^2^	Primary atom site location: structure-invariant direct methods
Least-squares matrix: full	Secondary atom site location: difference Fourier map
*R*[*F*^2^ > 2σ(*F*^2^)] = 0.048	Hydrogen site location: inferred from neighbouring sites
*wR*(*F*^2^) = 0.140	H atoms treated by a mixture of independent and constrained refinement
*S* = 1.00	*w* = 1/[σ^2^(*F*_o_^2^) + (0.0664*P*)^2^ + 0.427*P*] where *P* = (*F*_o_^2^ + 2*F*_c_^2^)/3
7545 reflections	(Δ/σ)_max_ = 0.003
433 parameters	Δρ_max_ = 0.26 e Å^−^^3^
13 restraints	Δρ_min_ = −0.26 e Å^−^^3^

### Special details

**Table:**

Geometry. Bond distances, angles etc. have been calculated using the rounded fractional coordinates. All su's are estimated from the variances of the (full) variance-covariance matrix. The cell esds are taken into account in the estimation of distances, angles and torsion angles
Refinement. Refinement of *F*^2^ against ALL reflections. The weighted *R*-factor *wR* and goodness of fit *S* are based on *F*^2^, conventional *R*-factors *R* are based on *F*, with *F* set to zero for negative *F*^2^. The threshold expression of *F*^2^ > σ(*F*^2^) is used only for calculating *R*-factors(gt) *etc*. and is not relevant to the choice of reflections for refinement. *R*-factors based on *F*^2^ are statistically about twice as large as those based on *F*, and *R*-factors based on ALL data will be even larger.

### Fractional atomic coordinates and isotropic or equivalent isotropic displacement parameters (Å^2^)

**Table:**

	*x*	*y*	*z*	*U*_iso_*/*U*_eq_	
F1	0.71950 (16)	0.40164 (7)	0.19658 (7)	0.0736 (4)	
F2	0.85905 (19)	0.38610 (9)	0.50961 (9)	0.0903 (5)	
N1	0.76753 (15)	0.21495 (9)	0.14701 (8)	0.0430 (4)	
N3	0.58511 (15)	0.13746 (9)	0.24705 (9)	0.0466 (4)	
C2	0.69990 (18)	0.20050 (10)	0.23469 (10)	0.0427 (4)	
C4	0.57872 (18)	0.10945 (10)	0.16402 (11)	0.0461 (4)	
C5	0.68894 (18)	0.15611 (10)	0.10096 (10)	0.0445 (4)	
C6	0.7308 (2)	0.15112 (14)	0.00357 (12)	0.0594 (6)	
C7	0.4597 (2)	0.03776 (13)	0.15297 (14)	0.0620 (6)	
C8	0.91789 (17)	0.26239 (10)	0.11653 (10)	0.0418 (4)	
C9	1.06498 (19)	0.23288 (11)	0.16966 (11)	0.0477 (5)	
C10	1.21032 (19)	0.27847 (12)	0.14079 (12)	0.0530 (5)	
C11	1.2127 (2)	0.35199 (12)	0.06008 (12)	0.0524 (5)	
C12	1.0649 (2)	0.38001 (12)	0.00716 (12)	0.0548 (5)	
C13	0.9174 (2)	0.33543 (12)	0.03523 (11)	0.0511 (5)	
C14	1.3728 (3)	0.40089 (18)	0.03046 (16)	0.0807 (8)	
C15	0.74677 (18)	0.25045 (10)	0.30559 (10)	0.0443 (4)	
C16	0.7547 (2)	0.34818 (11)	0.28531 (11)	0.0519 (5)	
C17	0.7926 (3)	0.39549 (13)	0.35225 (13)	0.0620 (6)	
C18	0.8220 (2)	0.34102 (14)	0.44250 (13)	0.0616 (6)	
C19	0.8149 (2)	0.24426 (13)	0.46788 (12)	0.0633 (6)	
C20	0.7775 (2)	0.19936 (12)	0.39909 (11)	0.0539 (5)	
F3	0.2298 (2)	0.39845 (7)	0.59771 (8)	0.0862 (5)	
F4	0.36352 (18)	0.38368 (9)	0.29162 (8)	0.0829 (4)	
N4	0.27463 (15)	0.21484 (9)	0.74141 (8)	0.0440 (4)	
N5	0.09244 (16)	0.13610 (9)	0.68227 (9)	0.0480 (4)	
C21	0.20702 (18)	0.19931 (10)	0.66138 (10)	0.0438 (4)	
C22	0.08604 (19)	0.10927 (11)	0.77972 (11)	0.0472 (4)	
C23	0.19611 (18)	0.15691 (11)	0.81797 (10)	0.0453 (4)	
C24	0.2371 (2)	0.15352 (14)	0.91762 (11)	0.0597 (6)	
C25	−0.0326 (2)	0.03794 (13)	0.82822 (14)	0.0631 (6)	
C26	0.42484 (18)	0.26301 (10)	0.74711 (10)	0.0436 (4)	
C27	0.4225 (2)	0.33889 (12)	0.78742 (12)	0.0513 (5)	
C28	0.5694 (2)	0.38378 (12)	0.79244 (12)	0.0563 (5)	
C29	0.7196 (2)	0.35333 (13)	0.75702 (11)	0.0539 (5)	
C30	0.7183 (2)	0.27746 (13)	0.71679 (12)	0.0567 (5)	
C31	0.5731 (2)	0.23190 (11)	0.71129 (11)	0.0510 (5)	
C32	0.8800 (2)	0.40155 (17)	0.76255 (16)	0.0783 (8)	
C33	0.25271 (19)	0.24827 (10)	0.56501 (10)	0.0452 (4)	
C34	0.2631 (2)	0.34591 (12)	0.53533 (11)	0.0556 (5)	
C35	0.3013 (3)	0.39309 (12)	0.44469 (12)	0.0640 (6)	
C36	0.3268 (2)	0.33864 (13)	0.38162 (12)	0.0587 (5)	
C37	0.3160 (2)	0.24260 (13)	0.40541 (12)	0.0604 (6)	
C38	0.2789 (2)	0.19759 (12)	0.49714 (11)	0.0527 (5)	
O1W	0.9309 (2)	0.00201 (13)	0.59088 (13)	0.0898 (7)	
O2W	0.4297 (2)	0.00074 (12)	0.40813 (12)	0.0872 (6)	
H6A	0.68540	0.09580	−0.00860	0.0890*	
H6B	0.85120	0.14680	−0.00280	0.0890*	
H6C	0.68290	0.20750	−0.04080	0.0890*	
H7A	0.34580	0.06540	0.15020	0.0930*	
H7B	0.47300	−0.01730	0.20590	0.0930*	
H7C	0.48360	0.01910	0.09580	0.0930*	
H9	1.06610	0.18300	0.22420	0.0570*	
H10	1.30890	0.25890	0.17680	0.0640*	
H12	1.06440	0.42930	−0.04790	0.0660*	
H13	0.81870	0.35490	−0.00080	0.0610*	
H14A	1.46100	0.35430	0.02430	0.1210*	
H14B	1.40560	0.43210	0.07730	0.1210*	
H14C	1.35380	0.44730	−0.02900	0.1210*	
H17	0.79790	0.46150	0.33670	0.0740*	
H19	0.83490	0.20940	0.53010	0.0760*	
H20	0.77270	0.13340	0.41540	0.0650*	
H24A	0.19330	0.09800	0.95860	0.0900*	
H24B	0.18710	0.20980	0.93260	0.0900*	
H24C	0.35740	0.15050	0.92620	0.0900*	
H25A	−0.01050	0.02150	0.89510	0.0950*	
H25B	−0.01700	−0.01840	0.80540	0.0950*	
H25C	−0.14670	0.06480	0.81540	0.0950*	
H27	0.32270	0.36000	0.81120	0.0620*	
H28	0.56740	0.43490	0.81980	0.0680*	
H30	0.81780	0.25640	0.69270	0.0680*	
H31	0.57500	0.18080	0.68380	0.0610*	
H32A	0.85530	0.45710	0.78560	0.1170*	
H32B	0.92750	0.42030	0.70080	0.1170*	
H32C	0.95930	0.35800	0.80470	0.1170*	
H35	0.30950	0.45890	0.42700	0.0770*	
H37	0.33340	0.20780	0.36060	0.0720*	
H38	0.27110	0.13180	0.51400	0.0630*	
H1WA	0.974 (4)	0.0404 (19)	0.614 (2)	0.1350*	
H1WB	0.9002 (18)	0.012 (2)	0.5427 (11)	0.1350*	
H2WA	0.471 (4)	0.0423 (18)	0.3654 (17)	0.1310*	
H2WB	0.4005 (19)	0.012 (2)	0.4516 (11)	0.1310*	

### Atomic displacement parameters (Å^2^)

**Table:**

	*U*^11^	*U*^22^	*U*^33^	*U*^12^	*U*^13^	*U*^23^
F1	0.1162 (9)	0.0530 (6)	0.0492 (6)	−0.0004 (6)	−0.0032 (6)	−0.0104 (4)
F2	0.1257 (11)	0.0887 (8)	0.0712 (7)	−0.0075 (7)	−0.0156 (7)	−0.0475 (7)
N1	0.0397 (6)	0.0493 (7)	0.0430 (6)	−0.0093 (5)	0.0014 (5)	−0.0155 (5)
N3	0.0430 (6)	0.0493 (7)	0.0487 (7)	−0.0082 (5)	0.0039 (5)	−0.0138 (5)
C2	0.0404 (7)	0.0456 (7)	0.0429 (7)	−0.0048 (6)	0.0016 (6)	−0.0127 (6)
C4	0.0415 (7)	0.0480 (8)	0.0512 (8)	−0.0057 (6)	−0.0010 (6)	−0.0167 (6)
C5	0.0402 (7)	0.0483 (8)	0.0482 (8)	−0.0058 (6)	−0.0019 (6)	−0.0178 (6)
C6	0.0597 (10)	0.0740 (11)	0.0536 (9)	−0.0172 (8)	0.0050 (7)	−0.0296 (8)
C7	0.0578 (10)	0.0644 (10)	0.0700 (11)	−0.0231 (8)	0.0033 (8)	−0.0236 (9)
C8	0.0384 (7)	0.0455 (7)	0.0449 (7)	−0.0083 (6)	0.0039 (6)	−0.0167 (6)
C9	0.0456 (8)	0.0493 (8)	0.0486 (8)	−0.0042 (6)	−0.0018 (6)	−0.0129 (6)
C10	0.0393 (8)	0.0667 (10)	0.0581 (9)	−0.0061 (7)	−0.0033 (6)	−0.0246 (8)
C11	0.0478 (8)	0.0619 (9)	0.0562 (9)	−0.0184 (7)	0.0099 (7)	−0.0274 (8)
C12	0.0591 (10)	0.0555 (9)	0.0494 (8)	−0.0146 (7)	0.0057 (7)	−0.0099 (7)
C13	0.0451 (8)	0.0572 (9)	0.0494 (8)	−0.0066 (7)	−0.0027 (6)	−0.0099 (7)
C14	0.0623 (11)	0.1094 (17)	0.0801 (13)	−0.0417 (11)	0.0171 (10)	−0.0329 (12)
C15	0.0424 (7)	0.0488 (8)	0.0444 (7)	−0.0048 (6)	0.0027 (6)	−0.0165 (6)
C16	0.0622 (10)	0.0497 (8)	0.0444 (8)	−0.0031 (7)	0.0029 (7)	−0.0133 (7)
C17	0.0801 (12)	0.0506 (9)	0.0606 (10)	−0.0072 (8)	0.0034 (9)	−0.0235 (8)
C18	0.0678 (11)	0.0704 (11)	0.0566 (10)	−0.0033 (8)	−0.0039 (8)	−0.0348 (9)
C19	0.0781 (12)	0.0663 (11)	0.0454 (9)	0.0036 (9)	−0.0087 (8)	−0.0168 (8)
C20	0.0630 (10)	0.0507 (8)	0.0480 (8)	−0.0011 (7)	−0.0011 (7)	−0.0135 (7)
F3	0.1543 (12)	0.0521 (6)	0.0568 (6)	0.0050 (6)	−0.0037 (7)	−0.0254 (5)
F4	0.1083 (9)	0.0851 (8)	0.0496 (6)	−0.0118 (7)	0.0139 (6)	−0.0059 (5)
N4	0.0427 (6)	0.0486 (7)	0.0430 (6)	−0.0066 (5)	0.0031 (5)	−0.0149 (5)
N5	0.0468 (7)	0.0501 (7)	0.0503 (7)	−0.0052 (5)	0.0013 (5)	−0.0181 (6)
C21	0.0440 (7)	0.0448 (7)	0.0449 (7)	−0.0011 (6)	0.0014 (6)	−0.0164 (6)
C22	0.0424 (7)	0.0486 (8)	0.0512 (8)	−0.0038 (6)	0.0041 (6)	−0.0136 (6)
C23	0.0412 (7)	0.0496 (8)	0.0453 (7)	−0.0040 (6)	0.0047 (6)	−0.0125 (6)
C24	0.0602 (10)	0.0737 (11)	0.0455 (8)	−0.0151 (8)	0.0042 (7)	−0.0134 (8)
C25	0.0589 (10)	0.0648 (11)	0.0675 (11)	−0.0197 (8)	0.0078 (8)	−0.0164 (9)
C26	0.0408 (7)	0.0478 (7)	0.0419 (7)	−0.0058 (6)	0.0016 (6)	−0.0103 (6)
C27	0.0447 (8)	0.0591 (9)	0.0556 (9)	−0.0072 (7)	0.0071 (7)	−0.0237 (7)
C28	0.0571 (9)	0.0599 (9)	0.0569 (9)	−0.0135 (7)	0.0013 (7)	−0.0216 (8)
C29	0.0450 (8)	0.0660 (10)	0.0463 (8)	−0.0119 (7)	−0.0028 (6)	−0.0045 (7)
C30	0.0404 (8)	0.0686 (10)	0.0569 (9)	0.0010 (7)	0.0054 (7)	−0.0100 (8)
C31	0.0491 (8)	0.0525 (8)	0.0517 (8)	0.0002 (7)	0.0050 (7)	−0.0148 (7)
C32	0.0528 (10)	0.1039 (16)	0.0762 (13)	−0.0268 (10)	−0.0046 (9)	−0.0139 (11)
C33	0.0451 (8)	0.0476 (8)	0.0441 (7)	−0.0022 (6)	−0.0002 (6)	−0.0144 (6)
C34	0.0728 (11)	0.0499 (8)	0.0474 (8)	−0.0005 (7)	−0.0043 (7)	−0.0196 (7)
C35	0.0867 (13)	0.0491 (9)	0.0543 (10)	−0.0092 (8)	−0.0042 (9)	−0.0090 (7)
C36	0.0619 (10)	0.0673 (10)	0.0435 (8)	−0.0055 (8)	0.0041 (7)	−0.0081 (7)
C37	0.0719 (11)	0.0641 (10)	0.0487 (9)	0.0036 (8)	0.0065 (8)	−0.0232 (8)
C38	0.0609 (9)	0.0492 (8)	0.0504 (8)	−0.0018 (7)	0.0034 (7)	−0.0179 (7)
O1W	0.0968 (12)	0.0877 (11)	0.0974 (12)	−0.0125 (9)	−0.0135 (9)	−0.0449 (10)
O2W	0.0945 (11)	0.0827 (10)	0.0774 (10)	−0.0101 (8)	0.0206 (8)	−0.0070 (8)

### Geometric parameters (Å, º)

**Table:**

F1—C16	1.3500 (19)	C10—H10	0.9300
F2—C18	1.357 (2)	C12—H12	0.9300
F3—C34	1.349 (2)	C13—H13	0.9300
F4—C36	1.355 (2)	C14—H14C	0.9600
O1W—H1WB	0.722 (16)	C14—H14A	0.9600
O1W—H1WA	0.82 (3)	C14—H14B	0.9600
O2W—H2WB	0.73 (2)	C17—H17	0.9300
O2W—H2WA	0.84 (3)	C19—H19	0.9300
N1—C5	1.3952 (19)	C20—H20	0.9300
N1—C8	1.4340 (18)	C21—C33	1.467 (2)
N1—C2	1.3640 (18)	C22—C23	1.357 (2)
N3—C2	1.3164 (19)	C22—C25	1.491 (2)
N3—C4	1.381 (2)	C23—C24	1.484 (2)
N4—C23	1.3938 (19)	C26—C27	1.377 (2)
N4—C26	1.4374 (19)	C26—C31	1.384 (2)
N4—C21	1.3668 (19)	C27—C28	1.387 (2)
N5—C21	1.318 (2)	C28—C29	1.390 (2)
N5—C22	1.382 (2)	C29—C32	1.508 (3)
C2—C15	1.473 (2)	C29—C30	1.378 (3)
C4—C5	1.356 (2)	C30—C31	1.381 (2)
C4—C7	1.493 (2)	C33—C34	1.381 (2)
C5—C6	1.484 (2)	C33—C38	1.389 (2)
C8—C13	1.376 (2)	C34—C35	1.374 (2)
C8—C9	1.383 (2)	C35—C36	1.369 (3)
C9—C10	1.383 (2)	C36—C37	1.357 (3)
C10—C11	1.376 (2)	C37—C38	1.377 (2)
C11—C14	1.509 (3)	C24—H24A	0.9600
C11—C12	1.384 (2)	C24—H24B	0.9600
C12—C13	1.389 (2)	C24—H24C	0.9600
C15—C16	1.379 (2)	C25—H25C	0.9600
C15—C20	1.393 (2)	C25—H25A	0.9600
C16—C17	1.379 (3)	C25—H25B	0.9600
C17—C18	1.366 (3)	C27—H27	0.9300
C18—C19	1.363 (3)	C28—H28	0.9300
C19—C20	1.377 (2)	C30—H30	0.9300
C6—H6C	0.9600	C31—H31	0.9300
C6—H6B	0.9600	C32—H32A	0.9600
C6—H6A	0.9600	C32—H32B	0.9600
C7—H7C	0.9600	C32—H32C	0.9600
C7—H7A	0.9600	C35—H35	0.9300
C7—H7B	0.9600	C37—H37	0.9300
C9—H9	0.9300	C38—H38	0.9300
			
H1WA—O1W—H1WB	125 (3)	C16—C17—H17	122.00
H2WA—O2W—H2WB	121 (3)	C18—C19—H19	121.00
C5—N1—C8	125.27 (12)	C20—C19—H19	121.00
C2—N1—C5	107.05 (12)	C19—C20—H20	119.00
C2—N1—C8	126.05 (12)	C15—C20—H20	119.00
C2—N3—C4	105.81 (12)	N5—C21—C33	124.47 (13)
C23—N4—C26	125.20 (12)	N4—C21—C33	124.54 (13)
C21—N4—C23	107.14 (12)	N4—C21—N5	110.98 (13)
C21—N4—C26	126.10 (12)	C23—C22—C25	129.00 (15)
C21—N5—C22	105.96 (13)	N5—C22—C25	120.43 (14)
N3—C2—C15	124.42 (13)	N5—C22—C23	110.56 (13)
N1—C2—N3	111.20 (13)	N4—C23—C22	105.36 (13)
N1—C2—C15	124.37 (13)	N4—C23—C24	123.10 (14)
N3—C4—C7	120.38 (14)	C22—C23—C24	131.53 (14)
N3—C4—C5	110.69 (13)	N4—C26—C27	120.98 (13)
C5—C4—C7	128.92 (15)	C27—C26—C31	120.14 (14)
C4—C5—C6	131.75 (14)	N4—C26—C31	118.88 (13)
N1—C5—C4	105.25 (12)	C26—C27—C28	119.81 (15)
N1—C5—C6	123.00 (13)	C27—C28—C29	120.84 (16)
N1—C8—C9	118.94 (13)	C30—C29—C32	120.69 (16)
N1—C8—C13	120.88 (13)	C28—C29—C30	118.17 (15)
C9—C8—C13	120.18 (14)	C28—C29—C32	121.14 (17)
C8—C9—C10	119.23 (15)	C29—C30—C31	121.73 (15)
C9—C10—C11	121.64 (15)	C26—C31—C30	119.32 (15)
C12—C11—C14	121.01 (17)	C21—C33—C38	120.50 (14)
C10—C11—C12	118.42 (15)	C34—C33—C38	116.59 (14)
C10—C11—C14	120.57 (16)	C21—C33—C34	122.82 (14)
C11—C12—C13	120.81 (16)	C33—C34—C35	123.51 (16)
C8—C13—C12	119.72 (15)	F3—C34—C33	118.53 (14)
C2—C15—C16	123.06 (13)	F3—C34—C35	117.94 (16)
C16—C15—C20	116.83 (14)	C34—C35—C36	116.79 (17)
C2—C15—C20	120.04 (14)	F4—C36—C37	119.22 (16)
C15—C16—C17	123.20 (15)	C35—C36—C37	122.91 (16)
F1—C16—C17	117.44 (15)	F4—C36—C35	117.87 (17)
F1—C16—C15	119.34 (14)	C36—C37—C38	118.70 (17)
C16—C17—C18	116.94 (18)	C33—C38—C37	121.48 (16)
F2—C18—C19	118.95 (16)	C23—C24—H24A	110.00
F2—C18—C17	117.96 (18)	H24A—C24—H24B	109.00
C17—C18—C19	123.10 (18)	H24A—C24—H24C	109.00
C18—C19—C20	118.38 (16)	H24B—C24—H24C	110.00
C15—C20—C19	121.56 (16)	C23—C24—H24B	109.00
C5—C6—H6A	109.00	C23—C24—H24C	109.00
H6B—C6—H6C	110.00	C22—C25—H25A	109.00
C5—C6—H6C	109.00	H25A—C25—H25B	110.00
H6A—C6—H6B	109.00	C22—C25—H25B	109.00
C5—C6—H6B	109.00	C22—C25—H25C	109.00
H6A—C6—H6C	109.00	H25B—C25—H25C	109.00
C4—C7—H7C	110.00	H25A—C25—H25C	109.00
H7A—C7—H7B	109.00	C28—C27—H27	120.00
C4—C7—H7B	109.00	C26—C27—H27	120.00
C4—C7—H7A	109.00	C27—C28—H28	120.00
H7A—C7—H7C	110.00	C29—C28—H28	120.00
H7B—C7—H7C	109.00	C29—C30—H30	119.00
C8—C9—H9	120.00	C31—C30—H30	119.00
C10—C9—H9	120.00	C26—C31—H31	120.00
C11—C10—H10	119.00	C30—C31—H31	120.00
C9—C10—H10	119.00	C29—C32—H32B	109.00
C11—C12—H12	120.00	C29—C32—H32A	109.00
C13—C12—H12	120.00	H32A—C32—H32C	110.00
C8—C13—H13	120.00	C29—C32—H32C	109.00
C12—C13—H13	120.00	H32A—C32—H32B	109.00
H14A—C14—H14B	109.00	H32B—C32—H32C	109.00
H14A—C14—H14C	109.00	C36—C35—H35	122.00
C11—C14—H14A	109.00	C34—C35—H35	122.00
C11—C14—H14B	109.00	C36—C37—H37	121.00
C11—C14—H14C	109.00	C38—C37—H37	121.00
H14B—C14—H14C	109.00	C37—C38—H38	119.00
C18—C17—H17	121.00	C33—C38—H38	119.00
			
C5—N1—C2—N3	−0.12 (17)	C14—C11—C12—C13	−179.34 (18)
C5—N1—C2—C15	−178.94 (14)	C10—C11—C12—C13	0.5 (3)
C8—N1—C2—N3	−166.06 (13)	C11—C12—C13—C8	−0.1 (3)
C8—N1—C2—C15	15.1 (2)	C2—C15—C16—C17	177.59 (17)
C2—N1—C5—C4	−0.17 (16)	C20—C15—C16—F1	−177.42 (14)
C2—N1—C5—C6	−179.31 (14)	C2—C15—C16—F1	−0.5 (2)
C8—N1—C5—C4	165.92 (13)	C2—C15—C20—C19	−177.35 (14)
C8—N1—C5—C6	−13.2 (2)	C16—C15—C20—C19	−0.3 (2)
C2—N1—C8—C9	52.4 (2)	C20—C15—C16—C17	0.7 (2)
C2—N1—C8—C13	−128.40 (17)	C15—C16—C17—C18	−0.4 (3)
C5—N1—C8—C9	−111.13 (17)	F1—C16—C17—C18	177.69 (17)
C5—N1—C8—C13	68.1 (2)	C16—C17—C18—F2	−179.81 (17)
C4—N3—C2—N1	0.34 (17)	C16—C17—C18—C19	−0.2 (3)
C4—N3—C2—C15	179.16 (14)	C17—C18—C19—C20	0.5 (3)
C2—N3—C4—C5	−0.45 (17)	F2—C18—C19—C20	−179.88 (15)
C2—N3—C4—C7	−179.74 (14)	C18—C19—C20—C15	−0.2 (2)
C23—N4—C21—N5	−0.03 (17)	N4—C21—C33—C34	−47.0 (2)
C23—N4—C21—C33	178.51 (14)	N4—C21—C33—C38	136.58 (16)
C26—N4—C21—N5	166.26 (14)	N5—C21—C33—C34	131.35 (17)
C26—N4—C21—C33	−15.2 (2)	N5—C21—C33—C38	−45.1 (2)
C21—N4—C23—C22	0.31 (17)	N5—C22—C23—N4	−0.49 (18)
C21—N4—C23—C24	179.64 (15)	N5—C22—C23—C24	−179.73 (16)
C26—N4—C23—C22	−166.13 (14)	C25—C22—C23—N4	−179.64 (16)
C26—N4—C23—C24	13.2 (2)	C25—C22—C23—C24	1.1 (3)
C21—N4—C26—C27	126.12 (17)	N4—C26—C27—C28	179.68 (14)
C21—N4—C26—C31	−53.9 (2)	C31—C26—C27—C28	−0.3 (2)
C23—N4—C26—C27	−70.0 (2)	N4—C26—C31—C30	−179.73 (14)
C23—N4—C26—C31	110.01 (17)	C27—C26—C31—C30	0.3 (2)
C22—N5—C21—N4	−0.27 (17)	C26—C27—C28—C29	0.2 (2)
C22—N5—C21—C33	−178.81 (14)	C27—C28—C29—C30	0.1 (3)
C21—N5—C22—C23	0.48 (18)	C27—C28—C29—C32	−179.77 (17)
C21—N5—C22—C25	179.72 (15)	C28—C29—C30—C31	−0.1 (3)
N1—C2—C15—C16	48.6 (2)	C32—C29—C30—C31	179.72 (17)
N1—C2—C15—C20	−134.62 (16)	C29—C30—C31—C26	−0.1 (3)
N3—C2—C15—C16	−130.11 (17)	C21—C33—C34—F3	0.0 (2)
N3—C2—C15—C20	46.7 (2)	C21—C33—C34—C35	−178.20 (17)
N3—C4—C5—N1	0.38 (17)	C38—C33—C34—F3	176.57 (15)
N3—C4—C5—C6	179.41 (16)	C38—C33—C34—C35	−1.6 (3)
C7—C4—C5—N1	179.59 (15)	C21—C33—C38—C37	177.67 (15)
C7—C4—C5—C6	−1.4 (3)	C34—C33—C38—C37	1.0 (2)
N1—C8—C9—C10	−179.85 (14)	F3—C34—C35—C36	−177.02 (17)
C13—C8—C9—C10	0.9 (2)	C33—C34—C35—C36	1.2 (3)
N1—C8—C13—C12	−179.83 (15)	C34—C35—C36—F4	179.56 (17)
C9—C8—C13—C12	−0.6 (2)	C34—C35—C36—C37	−0.1 (3)
C8—C9—C10—C11	−0.5 (3)	F4—C36—C37—C38	179.88 (15)
C9—C10—C11—C12	−0.1 (3)	C35—C36—C37—C38	−0.5 (3)
C9—C10—C11—C14	179.66 (18)	C36—C37—C38—C33	0.0 (2)

### Hydrogen-bond geometry (Å, º)

**Table:**

*D*—H···*A*	*D*—H	H···*A*	*D*···*A*	*D*—H···*A*
O1*W*—H1*WA*···N5^i^	0.82 (3)	2.17 (3)	2.987 (2)	177 (2)
O1*W*—H1*WB*···O1*W*^ii^	0.72 (2)	2.42 (2)	2.894 (3)	125 (2)
O2*W*—H2*WA*···N3	0.84 (3)	2.16 (3)	2.995 (2)	174 (2)
O2*W*—H2*WB*···O2*W*^iii^	0.73 (2)	2.42 (2)	2.910 (2)	126 (2)
C17—H17···F3^iv^	0.93	2.46	3.253 (2)	143

Symmetry codes: (i) *x*+1, *y*, *z*; (ii) −*x*+2, −*y*, −*z*+1; (iii) −*x*+1, −*y*, −*z*+1; (iv) −*x*+1, −*y*+1, −*z*+1.
